# A giant superficial myofibroblastoma involving the vagina and pelvis: A case report and review of the literature

**DOI:** 10.1016/j.radcr.2023.02.018

**Published:** 2023-03-07

**Authors:** Rennan Ling, Ren Li, Jinling Zhang, He Zhang

**Affiliations:** a Department of Radiology, Shenzhen People's Hospital (The Second Clinical Medical College, Jinan University; The First Affiliated Hospital, Southern University of Science and Technology), Shenzhen, 518020, China; b Department of Gynecology, Shenzhen People's Hospital (The Second Clinical Medical College, Jinan University; The First Affiliated Hospital, Southern University of Science and Technology), Road Donghubei No. 1017, Shenzhen, 518020, China; c Department of Radiology, Obstetrics and Gynecology Hospital, Fudan University, Road Shenyang No.128, Shanghai, 200020, China

**Keywords:** Giant, Myofibroblastoma, Mesenchymal tumor, Vagina

## Abstract

Superficial myofibroblastoma is a rare benign mesenchymal tumor that presents a challenge in accurate preoperative diagnosis because of its overlapping radiological and histological features. A 27-year-old woman presented with a history of increasing abdominal girth over the prior year and pelvic mass for 1 month. Imaging confirmed the presence of a giant well-circumscribed cystic-solid tumor involving both the extraperitoneal pelvis and vagina. After exploration and excision, superficial vaginal myofibroblastoma was diagnosed pathologically. The patient underwent surgical excision and had no postoperative complications at the 1-month follow-up. Imaging features and clinical reasoning can aid in differentiating superficial myofibroblastoma from more aggressive entities or malignant tumors and guide suitable and appropriate surgical approaches.

## Introduction

Superficial myofibroblastoma is a rare benign mesenchymal tumor that presents with a nodular or polypoid-like morphology in the lower genital tract in females; it occurs primarily in the vagina, rarely in the cervix, and occasionally in the vulva [1], [2], [3], [4], [5]. The pathological differential diagnoses are other genital mesenchymal tumors, such as fibroepithelial polyps, angiomyofibroblastoma, myofibroblastoma, cellular angiofibroma, and aggressive angiomyxoma (AA) [3].

To our knowledge, there have been 9 public reports of superficial vaginal myofibroblastoma (including a total of 35 cases) [1,^2^,[6], [7], [8], [9], [10], [11], [12]], of which only 3 involve incomprehensive imaging reports [6], [7], [8]. However, only 1 report [7] included a comparison of MRI features and histological features, and the detailed analysis was limited. Therefore, we present here a distinctive case of a giant tumor involving both the pelvis and vagina. By reporting imaging features and clinical reasoning, we aim to raise awareness about this lesion so that it may be considered in the differential diagnosis of lower female genital tract tumors, differentiate it from other more aggressive entities or malignant tumors, and guide appropriate clinical decisions, that is*,* how to avoid overly aggressive treatment and retain reproductive function, and management thereafter.

## Case

A 27-year-old woman, G0P0, presented with a 1-year history of increasing abdominal girth and a 1-month history of a giant pelvic mass. Occasionally, a small amount of vaginal bleeding occurred in the first 2 days after the menstrual period. Vaginal discharge, prolonged menstrual cycles and increased menstrual volume did not occur. She was not on any medication or hormonal therapy and did not have a history of gynecological therapy. Laboratory examination showed normal tumor biomarkers (ie, carcinoma antigen 19-9, 125, 153, and 742, carcinoembryonic antigen, alpha-fetoprotein, lactate dehydrogenase, human epididymis protein 4, and neuron specific enolase).

Gynecological examination revealed a giant pelvic lump (15 × 17 cm) that protruded into the vagina approximately 3 cm from the hymen. The displaced vaginal lumen was smooth, and the tumor was tender. The tumor pushed the cervix to the left side.

**Fig. 1:**
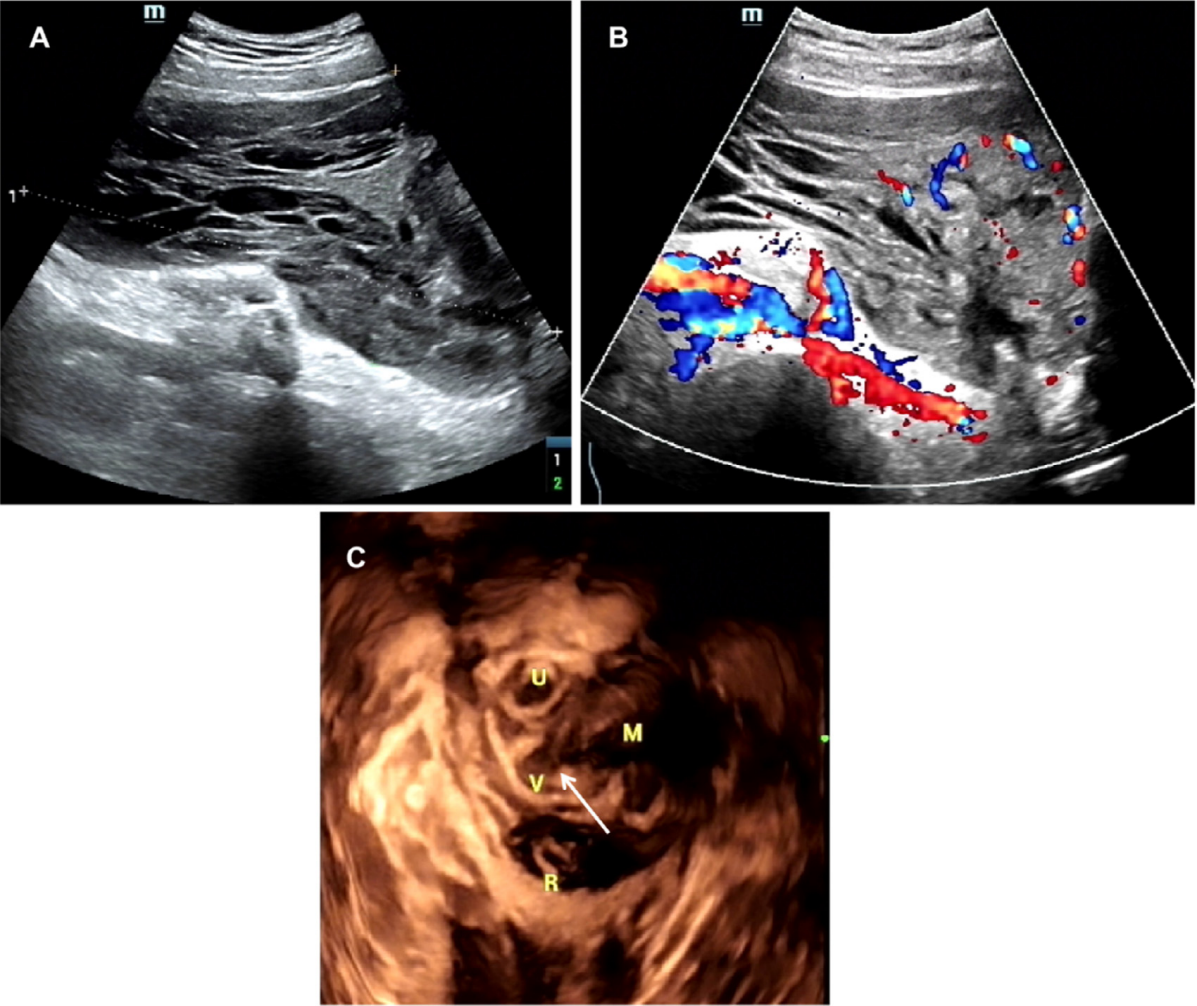
Ultrasonographic aspect of pelvic tumor and vaginal tumor. Abdominal ultrasound revealed a giant cystic-solid tumor (17 × 11.7 × 8.7 cm) in the pelvis, and (B) color Doppler flow imaging showed low blood flow in the tumor. (C) 3D ultrasound revealed a 3.5-cm vaginal defect (arrow) where pelvic tumor connected with vaginal tumor (3.6 × 6 cm). U, uterine; V, vaginal; M, mass; R, rectum.

Abdominal ultrasound and transvaginal ultrasound imaging detected a heterogeneous cystic-solid tumor (3.6 × 6 cm) in the left front wall of the vagina protruding into the pelvic wall (17 × 11.7 × 8.7 cm) (Fig. 1) with bladder, uterus, and cervix displacement posteriorly and to the right. Morphology was deformed by extrusion. Solid components were hypoechoic with multiple cord-like anechoic intervals. Color Doppler flow imaging showed low blood flow in the tumor. Pulsed wave (PW) Doppler showed a fluctuating arterial blood flow spectrum, and the resistive index (RI) value was 0.57.

**Fig. 2:**
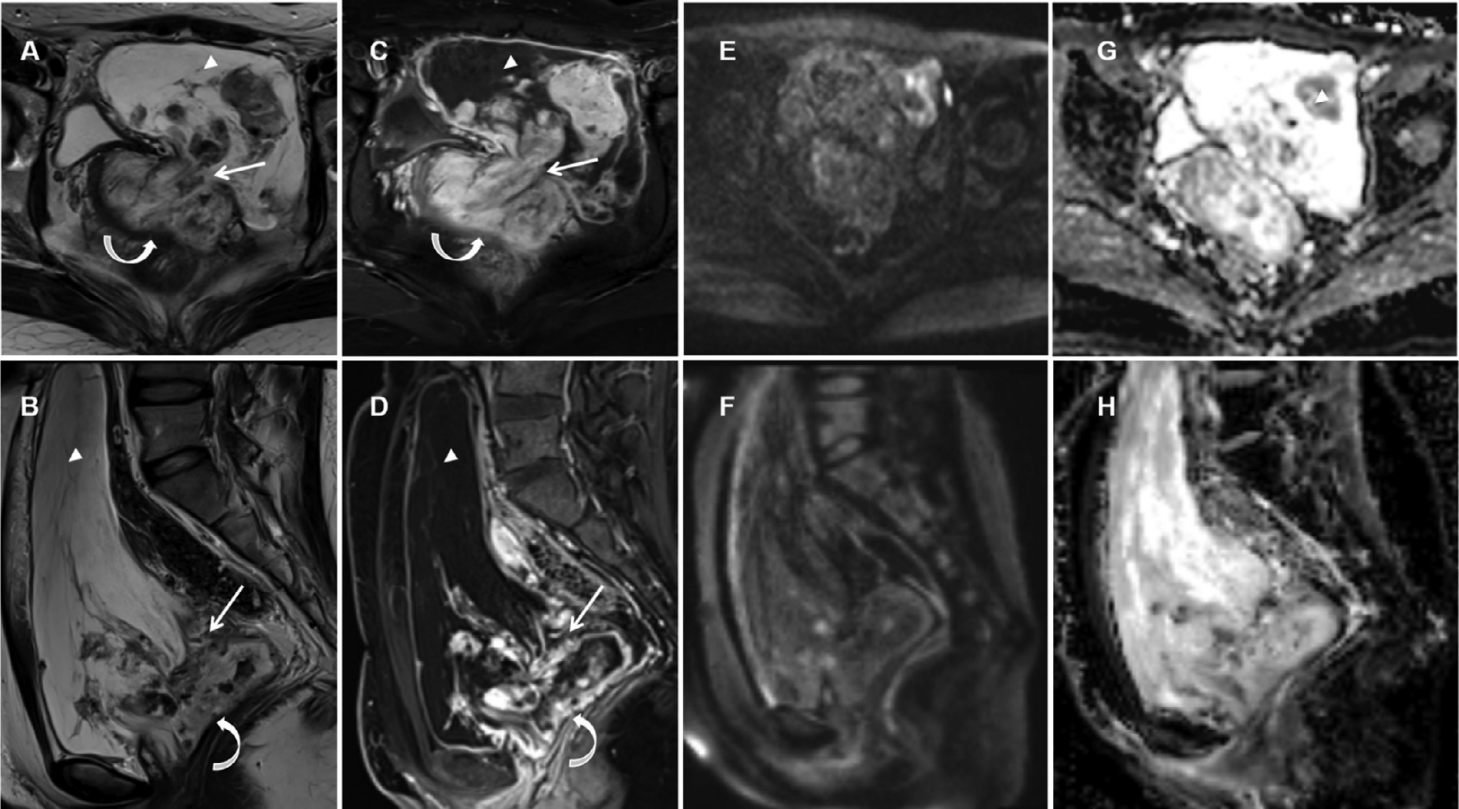
MRI aspect of pelvic tumor and vaginal tumor. (A) Transverse T2WI and (B) sagittal T2WI revealed a pelvic tumor (18 × 7.2 × 11.5 cm, arrowhead) and a vaginal tumor (6.5 × 2.6 × 6.2 cm, curved arrow) were connected at the defect (1.3 × 2.9 cm, arrow) of the vagina. Postcontrast fat-saturated (C) transverse and (D) sagittal T1WI showed the solid components intensely enhanced. (E-H) Diffusion-weighted images showed no restricted diffusion, and the mean apparent diffusion coefficient value was 1.492 × 10^−3^ mm^2^/s (arrowhead).

MRI confirmed a cystic-solid tumor located in both the extraperitoneal pelvis (18 × 7.2 × 11.5 cm) and the vaginal wall (6.5 × 2.6 × 6.2 cm), which connected at the defect (1.3 × 2.9 cm) of the left front wall of the vagina (Figs. 2A-D). The tumor was calabash-like, adapted in shape to the morphology of the extraperitoneal pelvic cavity. There was a well-circumscribed thickened fibrous capsule. Cystic components located at a superficial section*,* that is, tumor periphery, showed hypointensity on T1WI, mixed patchy hyperintensity on T2WI and no enhancement on a postcontrast fat-saturated T1WI. Solid components, located primarily close to the vagina, manifested multiple nodular and cord-like features with low T2 signal intensity and intermediate T1 signal intensity; these features were intensely enhanced similar to that of the uterus. A displaced vaginal lumen and integral vaginal mucosal layer indicated that the lesion could be differentiated from vaginal carcinoma. Furthermore, no restricted diffusion was observed, and the mean apparent diffusion coefficient value was 1.492 × 10^−3^ mm^2^/s, which could differentiate it from lymphoma, sarcoma and metastasis (Figs. 2E-H).

**Fig. 3:**
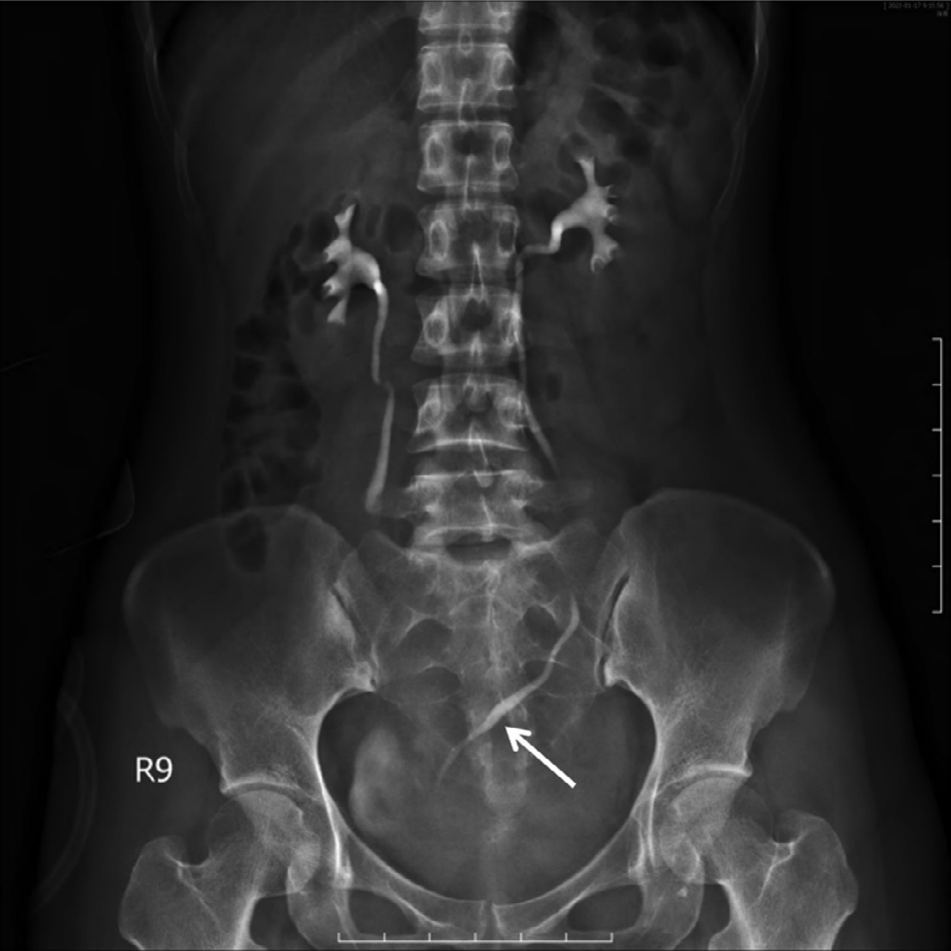
Intravenous urography examination showed the left ureter displaced to the midline region (arrowhead).

Intravenous urography examination revealed that the left ureter was displaced to the midline region (Fig. 3).

Ultimately, multidisciplinary team discussion was required preoperatively to guide appropriate management thereafter, and the presumptive diagnosis of benign mesenchymal tumor arising from vagina was made based on both imaging and clinical features.

**Fig. 4:**
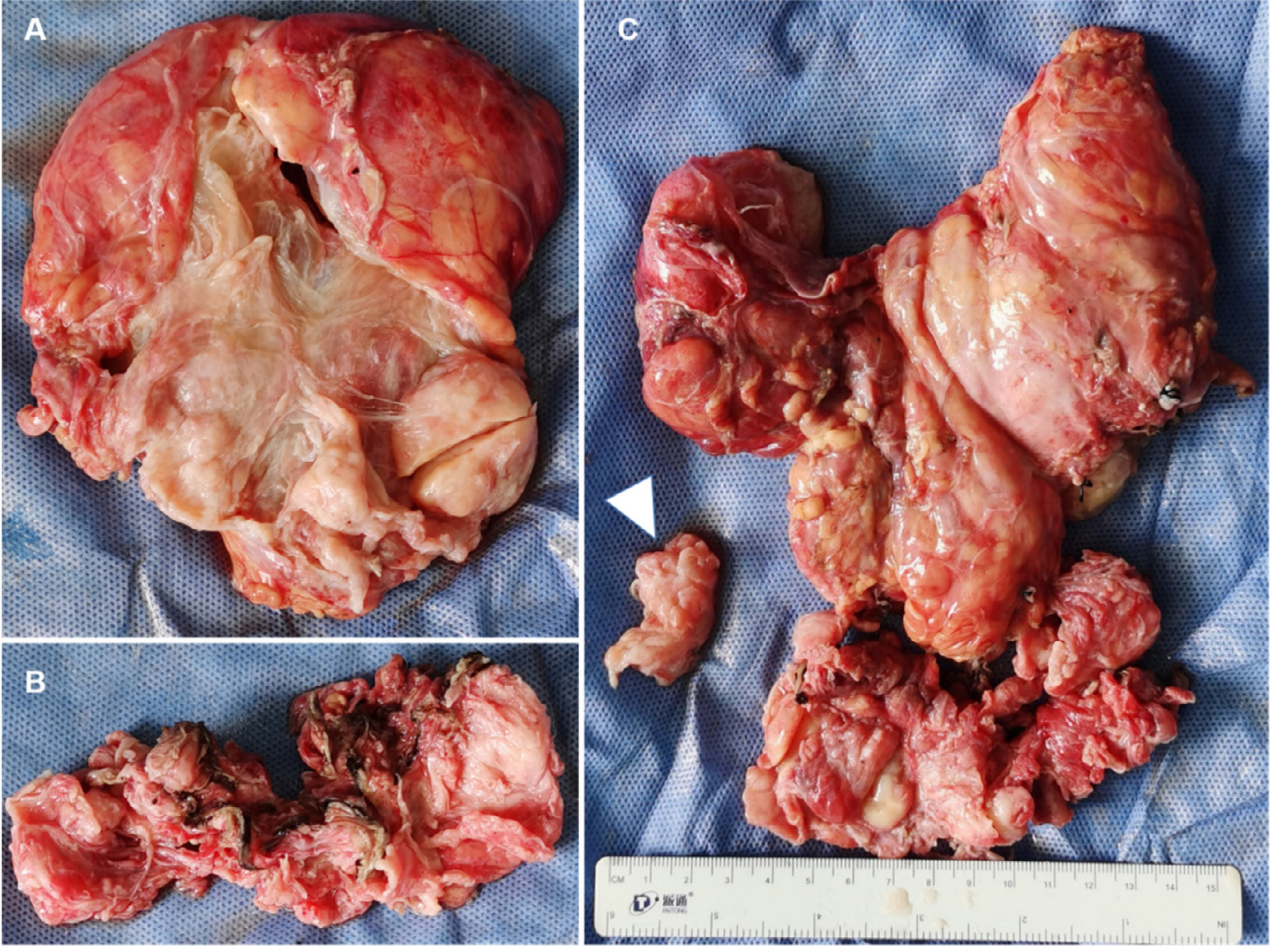
Gross examination view of the tumor. (A) Extraperitoneal pelvic tumor, (B) vaginal tumor, and (C) segmental vaginal wall (arrow) were excised.

Based on clinical reasoning, exploratory laparotomy was performed. Placement of bilateral ureteral stents was conducive to identification and was not injurious. Methylene blue was injected into the bladder to distinguish the boundary between bladder and pelvic tumors. After the bladder and bilateral ureters were separated from the pelvic tumor, the pelvic tumor was exposed completely. The tumor was tender and located in the extraperitoneal pelvis; it connected with the left front vaginal wall, and a thick stem was revealed. The vaginal tumor and pelvic tumor were connected at the thick stem. Moreover, the left section of the tumor extended to the pelvic sidewall. Ultimately, transabdominal extraperitoneal tumor resection was performed. Pelvic tumor, vaginal tumor, and segmental vaginal wall were completely removed en bloc (Fig. 4). As a key contributor to ensuring ovulation and pregnancy, the pelvic peritoneum and fascia were thoroughly repaired and closed. The residual left front wall of the vagina was so thin that it was repaired by intermittent suturing.

**Fig. 5:**
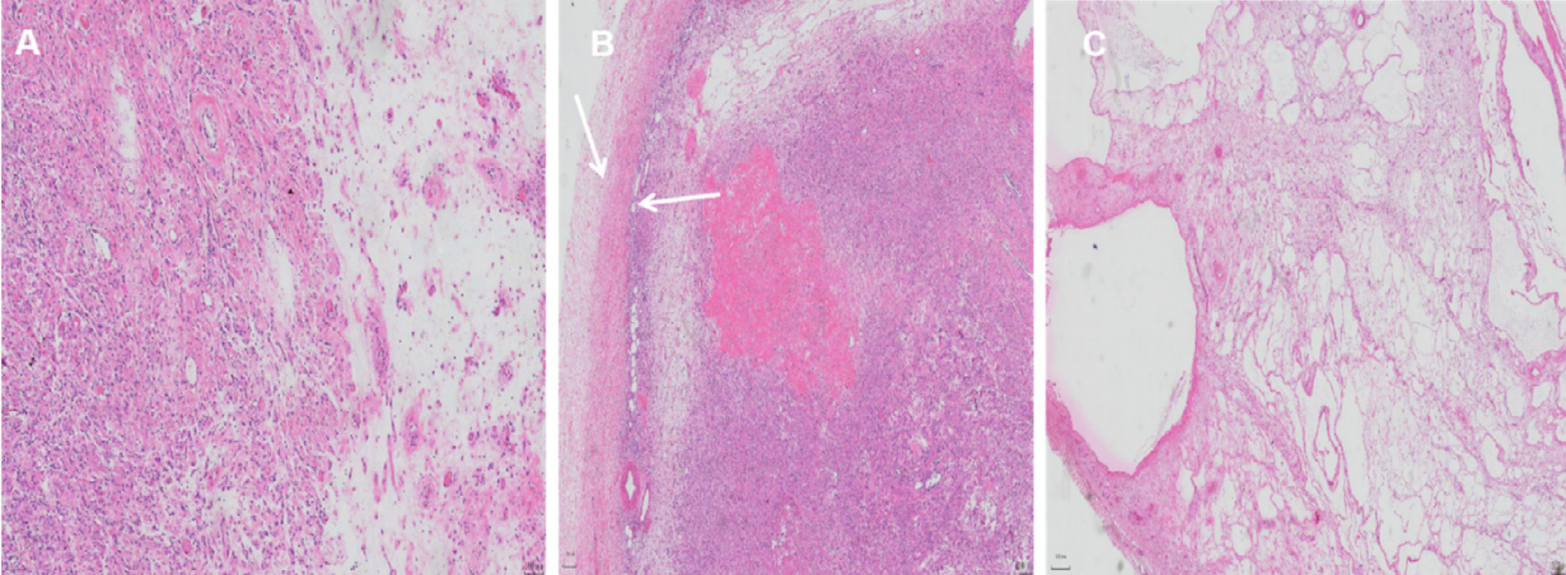
Microscopic examination view of the tumor. (A) Microscopic examination revealed 2 growth patterns of solid components (left side) and sparse areas (right side; scale bar: 100 μm). There was a thickened fibrous capsule, and (B) microscopic examination revealed a Grenz zone (area between arrows; scale bar: 200 μm). (C) Hypocellularity and marked mesenchyme edema foci contributed histologically to cystic components forming gross cystic morphology (scale bar: 500 μm).

Gross examination showed 2 tumor growth patterns: sparse areas and dense areas (Fig. 5A). The cavities of multicystic components, located superficially and peripherally, were filled with clear liquid and jelly-like matter, and the cystic walls were smooth with a thickness of 0.2-2.1 cm. The solid components showed multiple nodules of different sizes ranging from 1 to 4 cm in diameter. The nodules had various appearances that ranged in color from grayish white to grayish yellow and grayish red. Furthermore, there was a well-circumscribed thickened fibrous capsule, and microscopic examination revealed a Grenz zone identified deep within the surface (Fig. 5B). The sparse areas were punctuated by extensive collagen fibers and mesenchyme edema foci causing gross cystic morphology (Fig. 5C). In the dense area, the cells with small nucleoli were spindle- and tellite-shaped and arranged in storiform and fasciculated patterns. Small- to medium-sized thin-walled vessels were revealed. Mitosis was rare, and lymphocytes and mast cells were scattered.

Immunohistochemistry demonstrated that the tumor cells were positive for estrogen receptor, progesterone receptor, Vimentin, Desmin, cluster of differentiation 34 (CD34), smooth muscle actin (SMA), and Caldesmon and negative for cytokeratin (CK), epithelial membrane antigen (EMA), and soluble protein-100 (S-100 protein). The proliferation index, Ki-67, was approximately 3%. Ultimately, superficial myofibroblastoma was diagnosed pathologically.

The patient had no postoperative complications at the 1-month follow-up.

## Discussion

The pathogenesis of superficial myofibroblastoma is not well understood. The disease presents in women in the fifth through eighth decades of life [1]. However, nearly one-third of patients have a history of tamoxifen or hormone-replacement therapy, suggesting that hormones may possibly have a role in the development of the tumor [9]. There is no association with infection of human papilloma virus, Epstein–Barr virus, or human herpesvirus 8 [10]. In consideration of the location of the tumor within the vagina and the lack of evidence of recurrence and malignant potential after complete removal, complete local excision is advocated [13].

To date, there have been only 9 reports of superficial vaginal myofibroblastoma, in which a total of 35 cases have been described. A summary of these cases is presented in Table 1. The tumors range in size from 2 to 65 mm, presenting a gross morphology of polyp or mass. Polyp-like tumors can protrude into the vaginal lumen and can even extrude from the introitus on straining. There was no recurrence after the tumor was resected vaginally.

**Table 1:** Reported cases of superficial vaginal myofibroblastoma.

Report	Cases	Age (years)	Size of lesion	Remarks/complaints
1 (Laskin et al. [1])	12	57 (40-74)	1-6.5 cm	Presented as mass or polyp. One patient had a short history of vaginal discharge and mass prolapsed out of the vagina with urination. Another patient had a 2-week history of vaginal bleeding. In addition, others were asymptomatic
2 (Olinici et al. [2])	1	63	4 × 3.5 cm	Presented with a two year-history of a vaginal mass that enlarged gradually
3 (Tomita et al. [6])	1	42	22 × 22 × 32 mm	Abnormal vaginal bleeding for 3 months
4 (Smith et al. [7])	1	70	47 × 40 × 44 mm	A 5-month history of a painless vaginal mass, which extruded from the introitus on straining
5 (Atinga et al. [8])	1	50	24 × 31 × 24 mm	A history of prolonged menstrual bleeding and right iliac fossa discomfort
6 (Wang et al. [9])	4	55 (47-63)	1-5 cm	Three patients presented with mass that dropped out of the vagina. One patient was asymptomatic.
7 (Liu et al. [10])	1	59	20 mm	Presented with postcoital bleeding for 1 month
8 (Stewart et al. [11])	4	58 (40-61)	16-22 mm	Four patients were asymptomatic, incidental finding
9 (Ganesan et al. [12])	10	56 (23-80)	2-40 mm	One patient presented with postmenopausal bleeding. In addition, others were asymptomatic

Notably, our case represents the first report of a tumor involving both the pelvis and vagina. In this patient, the questions of how to strip the tumor integrally and how to protect both the ureter and bladder needed to be considered. The appropriate surgical approach to preserve fertility was crucial in treating a patient who had not been pregnant before. Multidisciplinary team discussion based on both imaging features and clinical features was required to guide appropriate clinical decisions. After comprehensive clinical reasoning and individual surgical procedures, the pelvis tumor and vaginal tumor were excised completely at the same time, avoiding overly aggressive treatment.

The essential differential diagnosis was AA, with massive mesenchyme edema and myxoid foci and imaging characteristics of "laminated" appearance and intense enhancement [14,15]. Given the infiltrative character, excision with a rim of surrounding normal tissue could possibly be insufficient and lead to local recurrences [8]. Immunohistochemistry also provided little help. The main distinguishing points were the location and the growth pattern. AA presents as a mass in a deep location and shows an aggressive growth pattern that can entrap surrounding tissues, such as fat tissue, blood vessels, neuron fibers, and muscles [14]. A recent report showed that HMGA2 is a useful marker of AA [16].

## Conclusion

To our knowledge, this is the first publicly reported case of a giant superficial vaginal myofibroblastoma involving both the pelvis and vagina. Due to overlapping radiological and histological features, making an accurate preoperative diagnosis of genital mesenchymal tumors is challenging. Imaging features and comprehensive clinical reasoning may aid in differentiating giant superficial vaginal myofibroblastomas from other more aggressive entities or malignant tumors and guide suitable and appropriate surgical approaches and management thereafter.

## Author contributions

RNL designed and performed the research study, collected data, took the pictures and wrote the original manuscript. RL contributed to the collected data. JLZ and HZ read and approved the final manuscript. All authors read and approved the final manuscript.

## Patient consent

A consent form was obtained from the patient and her family. Ethics committee approval is not needed.

## Appendix Supplementary materials

Image, application 1
