# Supplementary material for: A giant superficial myofibroblastoma involving the vagina and pelvis: A case report and review of the literature
Source: Radiol Case Rep. 2023 Mar 7;18(5):1862–7. doi: 10.1016/j.radcr.2023.02.018 (PMC10011681; doi:10.1016/j.radcr.2023.02.018)
Supplement: Supplementary file 1 [file mmc1.pdf]

INVOICE

Invoice# SC5BW3JNN

Balance Due
CNY0.00

美刊在线（北京）信息咨询有限公司
China

Invoice Date :	2023-01-27
Terms :	Due On Receipt
Due Date :	2023-01-27
Submission :	C5BW3JNN
Word Count :	2317
Title :	A giant superficial myofibroblastoma involving vagina and pelvis: a case report and review of the l…
Discounts Applied :	$40 (RBX40E)

Bill To
RENNAN LING
RENNAN LING

#	Item & Description	Rate	Discount	Amount
1	Standard Editing<br>Standard Editing	1,039.03	262.00	777.03

Sub Total	777.03
**Total**	**CNY777.03**
Payment Made	(-) 777.03
**Balance Due**	**CNY0.00**
